# Design of a Fuzzy Logic Evaluation to Determine the Ergonomic Risk Level of Manual Material Handling Tasks

**DOI:** 10.3390/ijerph19116511

**Published:** 2022-05-27

**Authors:** Martha Roselia Contreras-Valenzuela, Diego Seuret-Jiménez, Ana María Hdz-Jasso, Viridiana Aydeé León Hernández, Alma Nataly Abundes-Recilla, Eduardo Trutié-Carrero

**Affiliations:** 1 Faculty of Chemical Sciences and Engineering, Universidad Autónoma del Estado de Morelos, Avenida Universidad 1001, Chamilpa 62209, Morelos, Mexico; ana.hernandez@uaem.mx (A.M.H.-J.); vleon@uaem.mx (V.A.L.H.); alma.abundes@uaem.edu.mx (A.N.A.-R.); 2 Center for Research in Engineering and Applied Sciences, Universidad Autónoma del Estado de Morelos, Avenida Universidad 1001, Chamilpa 62209, Morelos, Mexico; eduardo.trutie@uaem.edu.mx

**Keywords:** ergonomics, fuzzy logic, risk evaluation, work task assessment

## Abstract

In this work, we propose a fuzzy inference as a decision support system built in the MATLAB Fuzzy Logic Designer for evaluating manual material handling risk conditions. The input variables for the fuzzy decision were: (1) the total time duration of the manual material handling in one shift of 450 min, with 3 h considered the maximal exposition time; (2) 25 kg as a maximal mass reference which should never be exceeded; (3) the repetitiveness of the manual material handling task through the shift considering as the maximal frequency of four lifts per min. Results of 135 earlier direct ergonomic evaluations made using the method proposed by the ISO 11228-1 were used as validator results, and called “expected results”. The experimentation intended to simulate an ergonomic evaluation in different boundary conditions of work and verify if the fuzzy interface could correctly replicate the results of the ergonomic evaluations. As validation, the list with the 135 expected results was compared against the evaluation made by the fuzzy logic interface, called “Work_Conditions”. From the comparison, only three evaluations (0.02%) differed with respect to the expected results. Consequently, it is concluded that the fuzzy interface can be used as a tool for automating the determination of manual material handling ergonomic risk levels, with great precision.

## 1. Introduction

Ergonomics is a science that combines other sciences (mechanics, physiology, mathematics, and physics, among others), taking a multidisciplinary character [1]. It integrates approaches with the principal objective of evaluating work systems and tasks for abating ergonomic risks present in them. The ergonomic purpose is to improve the workspaces, environments, and work methods with attention to human technical requirements [2,3]. The results of an ergonomic intervention are focused on the prevention of work-related illnesses and musculoskeletal disorders, considering the safety, health, and well-being of workers, without detriment to productivity and efficiency. This research work presents a fuzzy logic ergonomic assessment, built in the MATLAB Fuzzy Logic Designer for evaluating ergonomic risk in manual material handling. The fuzzy interface is an interactive computer system that helps specialists to solve differences in decision-making during ergonomic evaluations. Unfortunately, an ergonomic assessment involves degrees of decision; Panjaitan and Bin [4], categorize a minimum of three types of classification of ergonomic assessments, and Grooten and Johanssons have identified at least 19 different ergonomic observation methods, all of which may identify uncertain conditions and involve balancing trade-offs [5]. Hence, for assessing a single task, there can be as many different results as the methods used. From an ergonomic point of view, there are two kinds of risk evaluation [5,6,7]: the simple risk assessment or general risk estimation (GRE), where the evaluation depends mainly on analysts’ expertise and point of view, which turns subjective the results and implementation, and the detailed risk-assessment or evaluation (DRE) which include a standardized method of evaluation based on occupational biomechanics. The ISO 11228-3 standard [7] includes a non-exhaustive list of the most currently used methods for GRE of repetitive movements/exertions at high frequency, such as RULA, REBA, OWAS, EPR, and JSI, among others [8,9,10,11,12]; the standard considers most of them as empiric and not tailored for DRE. For manual material handling, the most well-known GREs are the MAC tool (manual handling assessment charts) [13] and the RAPP tool (risk assessment of pushing and pulling) [14]. Both tools do not comprise a suitable and sufficient risk assessment; if the result is vague, it will be necessary to implement a DRE. Therefore, these kinds of methods are not recommended for our purposes.

The case of detailed risk assessments (DRE) is more suitable for our investigation. An example of DRA is the revised OCRA checklist method [15], which is included in the international standards EN 1005-5 [16] and ISO11228-3 [7]. Hence, a careful selection of the method for ergonomic evaluation must be performed to assure accuracy in the results. In a working system where a worker develops tasks that include manual material handling, the ergonomic standard ISO 11228-1 [17] proposes a framework for assessing task performance, using three elements: mass of the object, lifting frequency, and exposition time, by placing an equal level of importance on each, assuring high precision in risk-level identification. The standard provides a phased method for estimating the health risks of manual lifting and carrying; through the evaluation of the frequency of carrying, cumulative mass per minute or hour until 8 h, and the exposition time; the method is clear and concise. 

However, in manual material handling, some complex tasks and activities cannot easily be evaluated because the results of their risk evaluation are vague (the risk difference during handling two boxes stacked one on top of the other on a pallet), i.e., fuzzy boundaries characterize the risk level. For example, when the results of the assessment are located in the boundary between safety conditions or high risk, the decision making, which is about determining the ergonomic risk level, could produce controversy among analysts. To resolve this discrepancy, it is necessary to determine a quantitative way to describe borderline cases. However, this is not always easy. Hence, designing one application to help to resolve differences of opinion is necessary. Therefore, in this context, one of the most successful soft computing techniques which have been used in multiple applications is Fuzzy Logic (FL), which takes the uncertainty from its inputs and compromises with it in a condition that the results are not affected by this variability; therefore, the result is precise [18]. FL models the vagueness present in the language when describing some phenomena that do not have sharply defined boundaries [19]. 

A systematic investigation review for recent years has identified that scholars are focused on resolving vagueness in ergonomic principles in three different combinations:

1. Ergonomics design (ED) using FL;
2. Ergonomic intervention (EI) and fuzzy approaches (FA);
3. Ergonomic risk evaluation (ERE) and FL.

Examples of the first combinations included the formulation of ergonomic diagnosis of a multi-agent manufacturing system proposed by Pacholski [20]; using the relations between parameters from ergonomic diagnosis as input data for a fuzzy set (FS), the results made up the basis of the soft inference concerning to the analysis of design, the ergonomic quality parameters reflecting the relation with human–machine interfaces. Aluclu et al. [21] proposed two FL-based models for noise control in industrial workplaces. The first model comprises linguistic rules and acoustical features of all materials used in any workplace and the second model deals with atmospheric parameter interactions with noise; the rules were determined by considering formal stand. As a result, models can be used for noise control in any workplace and help the designer in the planning stage of a workplace. Pancardo et al. [22] proposed an FL-based personalized method to classify perceived exertion in workplaces using a wearable heart-rate sensor; the research aim was to provide personalized follow-up on efforts carried out by workers during task execution, and the results facilitate the decision-making of supervisors regarding the worker allocation in the appropriate job to prevent accidents. Ani et al. [23] proposed a decision support system (DSS) using an ergonomic approach for detecting driving fatigue, through the development of a driving fatigue strain index using an FL-membership function; the result is a graphical user interface that offers solutions and recommendations to minimize the number of road accidents in Malaysia. 

Contributions in the field (2) have been achieved by publications that grouped different FA, i.e., the goals determined by Hamadi et al. [24], who involve the fuzzy Delphi method and EI. The study aimed to identify and determine the most important goals of EI from the perspective of experts from the Iranian industry. Another study on this subject called the design of the evaluation model for total EI with FA was proposed by Abarqhouei et al. [25]. The investigation explains how a suitable relationship between staff and work can be achieved, where staff can have maximum productivity and production through the development of a theory for the guidance of EI and evaluation processes with the help of FA. 

Finally, in the field (3), Bockelman [26], proposed a site-based ergonomic assessment of acoustics in school settings and an FL metric; she tested noise on two school campuses. The results of the study were analyzed, and they reflect a noise level that exceeds the ideal learning conditions and potentially endangers hearing. A study by Galabchi et al. [27] proposed an FL approach to posture-based ergonomic evaluation tools to describe the FL modeling for the scoring of RULA assessment systems and its application to modular construction shops. Hybrid fuzzy logic modeling and software for ergonomics assessment of biotechnical systems were proposed by Al-Kasasbeh et al. [28], which includes a method of synthesis of hybrid fuzzy decision rules groups for the analysis of the data structure, especially algorithm exploratory analysis based on FA. Cruz et al. [29] and Nunes [30,31]. The first one was called the fuzzy logic and RULA method for assessing the risk of working. Its aim was to minimize the valuation work for every operator through three fuzzy sets (for arm, forearm, and wrist). The second research study comprised an ergonomic analysis tool: a fuzzy expert system called FAST ERGO_X-A designed to support ergonomic auditing activities related to musculoskeletal disorders; the tool helps with the identification, assessment, and control of risk factors present at workstations. Some investigations are based on designing fuzzy assessments of environments; however, this kind of research has no ergonomic purposes. For example, Colella et al. [32] proposed a fuzzy inference system for the assessment of indoor air quality in an operating room to prevent surgical-site infections. 

The dissertation established above involves uncovered factors and attributes for ergonomic risk evaluations using FL that seem to be crucial for an ergonomic risk assessment in manual material handling. The number of publications dealing with this process perspective remains low. No work has been found in the literature that specifically relates a combination of Fuzzy Logic with ISO 11228 standard, as this work proposes. The aim to reduce vagueness using Fuzzy Logic applied in ergonomic decisions is to improve the method of developing risk assessment with updated purposes, through a solid data evaluation of its rules, incorporating a technique that minimizes differences of point of view during the decision-making. 

In this work, we propose a fuzzy logic ergonomic assessment (FzEA), as a decision support system (DSS) built in the MATLAB Fuzzy Logic Designer. Its objective is to evaluate tasks that include manual material handling and define a level of risk and the severity of the impact on the workers’ health, evaluating risk conditions that lead to musculoskeletal disorders. The fuzzy interface is an interactive computer system that helps specialists to solve differences in decision-making during ergonomic evaluations. The aim is to reduce the vagueness of the results using fuzzy logic applied to ergonomic decisions. 

## 2. Materials and Methods

### 2.1. Context of Manual Material Handling Accordingly with ISO11228-1[17]

To design and test the proposed FzEA, three conditions of manual handling tasks were considered: the mass of the object to be manipulated; the duration of the task, defined as the time of exposition; and the lifting frequency. The standard recommends that the limits of maximal mass reference and frequency must have the highest priority. The cumulative mass is estimated as a product of mass and repetitiveness of carrying, defined in the Equation (1). The mass reference of 25 kg and the maximal frequency of 15 manual lifting per minute should never be exceeded. The limits should apply depending on the gender and age of the worker [17], and in the case of pregnant women, the Mexican low establishes the reduction of the mass weight to 10 kg [6]. However, from the FL point of view, it was necessary to establish general limit conditions; thus, the ISO 11228-1 parameter was used: 3 kg as low mass and 25 kg as higher mass.

(1) (m·f),

**Definition** **1.** *Manual handling is any activity requiring the use of human force to lift, lower, carry, or otherwise move or restrain an object.*

**Definition** **2.** *Reference mass, mass considered appropriate for use with an identified user population during the application of the risk assessment.*

**Definition** **3.** *Repetitive handling, handling an object more than once every 5 min.*

**Definition** **4.** *Frequency of lifting actions; define the number of actions per unit of time.*

**Definition** **5.** *Task duration (time of exposition); duration of manual handling that a static working posture is maintained.*

The limits are not simple multiplications because the risks are qualitatively different in terms of time.

### 2.2. Step (1) Determination of Risk Levels (Fuzzy Choices)

During the experimental activities for the FzEA design, three levels of risk (low, medium, and high) were determined, and they were used as fuzzy choices. The impacts on the workers’ health, defined by the ISO 11228-1, are:

- Low risk (long term): Conditions present in carrying and lifting tasks that do not generate work-related illness over a long time;
- Medium risk (medium-term): Conditions present in carrying and lifting tasks that generate work-related illness, in a medium amount of time;
- High risk (short term): Conditions present in carrying and lifting tasks which generate work-related illness in a short time.

It is important to consider that, despite a low risk level being defined individually for each level of risk, the combination of different levels of conditions could change the final risk level. For example, only one lift during the shift is considered low frequency in a short exposition time, but if the mass is near 25 kg, the task could be dangerous for workers without good health. Another example would be a task in which workers manipulate 3 kg (low risk) 1800 times (high risk) in only 30 min (low risk), which implies 5400 kg of accumulated mass; that is, half of the mass to be handled during an 8 h shift. This kind of task can lead to developing a musculoskeletal disorder in a medium amount of time. A case in which the three parameters are low is unlikely. However, if applicable, the task will be identified as safe. 

### 2.3. Step (2) Definition of Ergonomic Parameters for Fuzzy Sets

#### 2.3.1. Ergonomic Parameters and Risk Level for the Time of Exposition 

The task duration considered as the time of exposition was divided into three sets, as shown in Table 1, which defines the limits for each risk level. The fuzzy limits for low and medium risk are between 60 min and 80 min, while the fuzzy limits for medium and high risk are between 100 min to 120 min. Limits of 450 min (8 h) by shift should not be exceeded.

#### 2.3.2. Ergonomic Parameters and Risk Level for the Mass of the Object 

The assignment of risk level for the mass of the object to be manipulated is shown in Table 2. The fuzzy limits for low and medium risk are between 7 kg and 10 kg, while the fuzzy limits for medium and high risk are between 13 kg and 15 kg. A cumulated mass of 10,000 kg in a shift of 8 h should not be exceeded, with a maximal reference mass of 25 kg.

Assumptions for Table 2: even though the ISO11228-1 determine a reference mass for different population (from 5 kg to 40 kg), we did not use all the range of mass references included in it (please refer to Table C.1 in [17]), because we were limited by the Mexican standard NOM 036-1:2018, due to its content is smaller (see Table A2). Therefore, a combination of both standards was made within the FzEA. For example, in Table 2, low risk includes the range 0 kg to 10 kg (5 kg and 10 kg from the ISO11228-1; however, medium risk starts from 7 kg to 15 kg, as is established in NOM 036-1 as well, as it contains 15 kg from ISO11228-1. Additionally, 15 kg is the maximal mass reference for Females in México; the high risk starts at 13 kg. Concerning recommended limits for cumulative mass related to carrying distance, we considered a distance of 1 m, with a maximal cumulative mass of 10,000 kg/8 h (please refer to Table 1 in [17]).

#### 2.3.3. Ergonomic Parameters and Risk Level for Frequency of Handling 

The recommended limits for the mass of the object relative to lifting frequency, see Equation (1) should be in the range of 5 kg × 15 times/min to 25 kg × 1 time/min. Table 3 shows the assignment of risk level for frequency of manipulation. The fuzzy limits for low and medium risk are between 600 movements and 700 movements, while the fuzzy limits for medium and high risk are between 900 movements and 1800 movements.

### 2.4. Step (3) Define Fuzzy Element for the FzEA in MATLAB Fuzzy Logic Designer

#### 2.4.1. The MATLAB Fuzzy Logic Designer 

The Fuzzy Logic Designer (FLD) is a Fuzzy Interface System (FIS) developed by MATLAB. The FIS is an intelligent system for analyzing, designing, and simulating systems based on fuzzy logic, which includes common methods such as fuzzy clustering and adaptive neuro-fuzzy learning. The toolbox allows model system behaviors using simple logic rules and then implements these rules in a FIS [33]. In this investigation, the FLD was applied in a particular field of ergonomics to model the high complexity and uncertainty that characterizes the decision-making during ergonomic assessments; however, it can apply to many domains. Its applications transfer the ergonomic knowledge into intelligent and automatic models using linguistic terms. The FIS used for building the FzEA is integrated with five components:

- The fuzzy logic designer editor, where the input and output variables are defined;
- The membership function editor, where input variable values are implemented to their membership function to determine the degree of truth of each premise;
- The rule editor, where experts’ experience is processed as fuzzy rules. The membership functions and variables of input and output are defined by the expert according to his experience.
- The rule viewer is a mapping of a fuzzy subset for each output variable of the rule. Its process of decision-making comprises evaluating a set of alternatives to relevant objectives and restrictions. The fuzzy sets consisted of objectives and restrictions defined in a linguistic form. The decision-making will be determined considering their joint or aggregate consideration, and it is similar to human analysis. Decisions are inferred and based on the calculation of the degree of truth in their premise.
- The surface viewer is a graphical interface that shows the linear relationship between variables.

#### 2.4.2. Fuzzy Sets 

A fuzzy set is defined as one in which its elements belong to it with a certain degree of membership *µ* [18] defined as a number *x* between 0 and 1 (interval [0, 1]), and are used to process uncertainty and characterize knowledge through rules. Thus, the concept of a fuzzy set associated with a certain linguistic value, defined by a word, adjective, or linguistic label *A*, is introduced. Then, it can be said that a fuzzy set *A* is defined as a membership function that links or matches the elements of a domain or universe of discourse *X* with elements of the interval [0, 1]; for each fuzzy set, a membership or inclusion function *μA(x)* is defined, which represents the degree to which a value for the variable *x* is included in the concept represented by the label *A*. The closer *A(x)* is to value 1, the greater the membership of object *x* to set *A*. Membership values vary between zero (does not belong at all) and one (total membership), so a fuzzy set is a class of objects with continuous degrees of membership [34].

The fuzzy logic allows the interpretation of data with predefined linguistic variables using conditional operators defined as IF-THEN rules [32], written as:

IF situation 1 AND situation 2 THEN the decision

where the situations represent the premise in fuzzy terms connected by fuzzy operators, while the output is the decision expected. Therefore, fuzzy logic defines the inferential mechanism needed to reach the output value related to the work condition and its ergonomic risk level. The inferential mechanism is provided by the main ergonomic parameters through the FLD.

**Figure 1:**
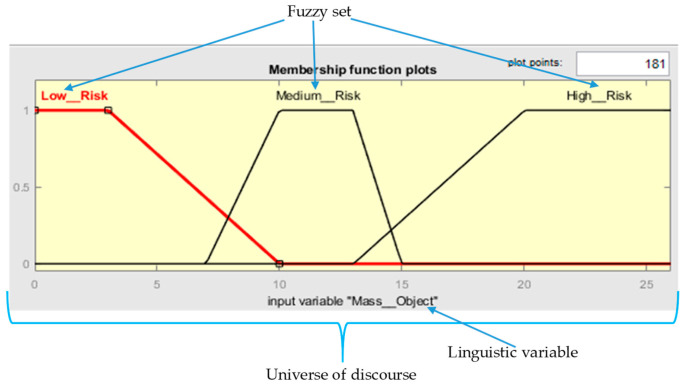
The representation made by the Fuzzy Logic Designer of the universe of discourse, linguistic values, fuzzy sets, and membership function.

Therefore, the universe of discourse is the range of values that can be taken by the elements that have a property expressed by the linguistic variable; for example, the kilograms that can be manipulated by a worker. A linguistic value refers to the different classifications performed on the linguistic variable. For example, the risk level is Low, Medium, and High. A membership function is an application that links every element of a fuzzy set to the degree it belongs to the associated linguistic value. A fuzzy set can also be represented graphically as a function, especially when the universe of discourse X (or underlying domain) is continuous (not discrete), as can be observed in Figure 1.

#### 2.4.3. Rules for the Fuzzy Ergonomic Assessment (FzEA)

**Figure 2:**
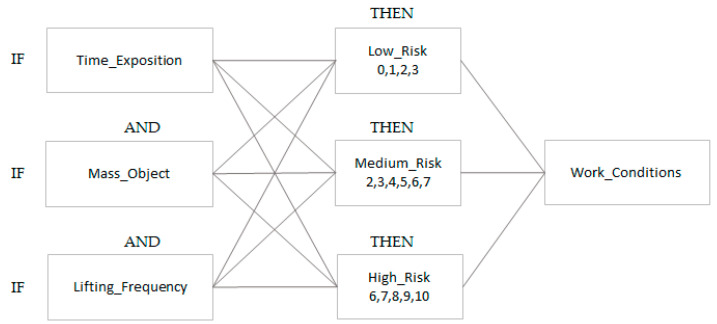
Grid diagram of work conditions (variables) for level (choices).

The goal of the FzEA is to predict the different grades of risk related to work conditions during manual material handling and identify the level present in the task. The linguistic model was built on fuzzy IF-THEN rules, where input variables were defined as linguistic variables (Time_Exposition, Mass_Object, lifting_Frequency); the consequent sentences were also defined as linguistic variables (Low_Risk, Medium_Risk, and High_Risk); see Figure 2. Therefore, it is an intuitive model which represents the specialists’ knowledge by determining the Work_Conditions in a range of 0 to 10 points, and standard parameters from the ISO 11228-1 in the system. A decision matrix was defined using the group of fuzzy choices. The results have to be decision-making to define the risk level for each work condition, where the limits of maximal mass have the highest priority, followed by frequency. Therefore, 27 rules were determined, and are shown in Table 4.

### 2.5. Step (4) Built the FzEA in MATLAB Fuzzy Logic Designer

**Figure 3:**
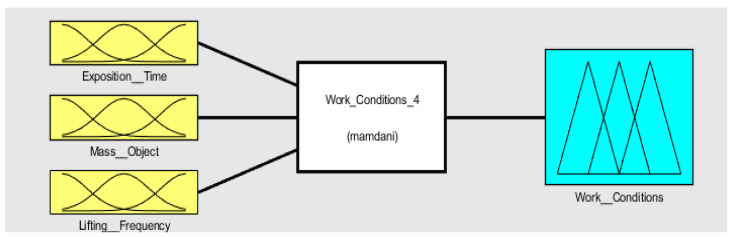
Default Mamdani-type inference display.

Using the default Mamdani-type inference display (see Figure 3), we started by adding the three variables of input identified as “Exposition_Time”, “Mass_Object”, and “Lifting_Frequency” and the output variable “Work_Conditions”. The FSI interface uses the Mamdani linguistic model in three steps: fuzzification, inference, and defuzzification. 

#### 2.5.1. Fuzzification

##### Exposition_Time

**Figure 4:**
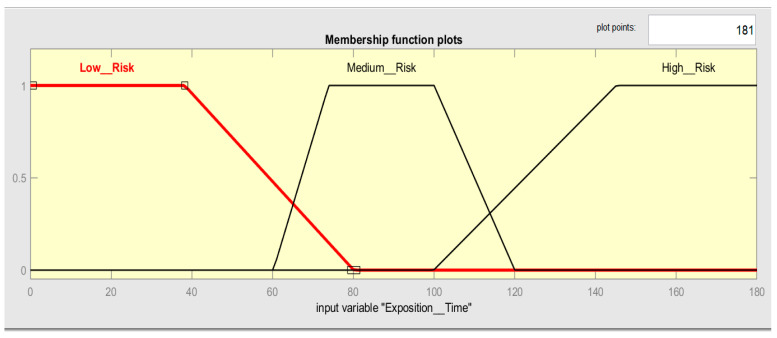
Membership function plots of Exposition_Time.

For the input variable called Exposition_Time, the membership of three trapezoidal fuzzy sets has been built considering the information presented in Table 1. The time below 30 min has a membership set to 1 and, with all certainty, is a low risk (see Figure 4); however, to create a trapezoidal membership function, it is necessary to create a decreasing ramp between 30 min and 80 min with the aim of gradually smoothing the degree of belonging in a range from 0 to 1, creating a transition from low risk to medium risk. The time above 150 min is certainly a high risk; it has a membership set to one. In the opposite case, to create a trapezoidal membership function, it is necessary to build an increasing ramp between the 100 min to 150 min, with the aim of gradually increasing the degree of belonging in a range from 0 to 1. The times in the range of 60 min to 120 min should receive the same treatment, increasing the ramp from 60 min to 80 min and decreasing the ramp from 100 min to 120 min. Times between 60 min and 80 min and 100 min to 120 min represent the vagueness of the decision. Table 5 shows the risk ranges considered with the respective fuzzy sets.

##### Mass_Object

**Figure 5:**
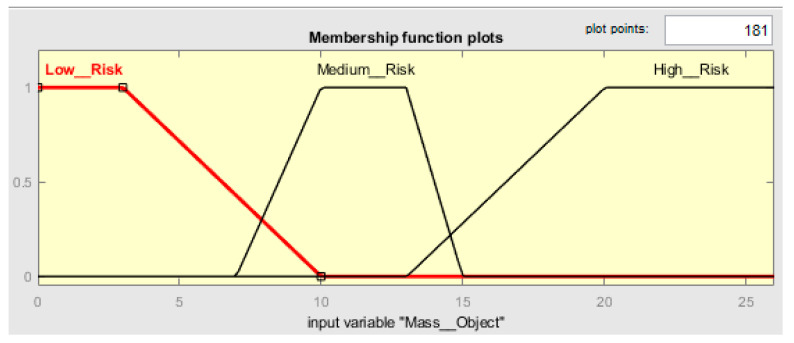
Membership function plots of Mass_Object.

For the input variable called Mass_Object, the membership of three trapezoidal fuzzy sets has been built considering the information in Table 2. The mass below 3 kg has a membership set to one and, with all certainty, is low risk (see Figure 5). For mass below 3 kg, normally the assessment of a task is made using the standard ISO 11228-3. To create a trapezoidal membership function, it is necessary to create a decreasing ramp between 3 kg and 10 kg to gradually smooth the degree of belonging in a range from 0 to 1, creating a transition from low risk to medium risk. The mass above 20 kg is certainly high risk; it has a membership set to 1. Meanwhile, 25 kg is recommended as the maximal mass to be manipulated for the adult male and 10 kg. To create a trapezoidal membership function, it is necessary to build an increasing ramp between 13 kg to 20 kg, to gradually increase the degree of belonging in a range from 0 to 1. The mass in the range of 6 kg to 15 kg should receive the same treatment, increasing the ramp from 6 kg to 8 kg and decreasing the ramp from 13 kg to 15 kg. Masses between 7 kg and 10 kg and 13 kg and 15 kg represent the vagueness of the decision. Additional factors may be considered, such as age and gender. Table 6 shows the risk ranges considered with the respective fuzzy sets for a distance of 1 m.

**Figure A1:**
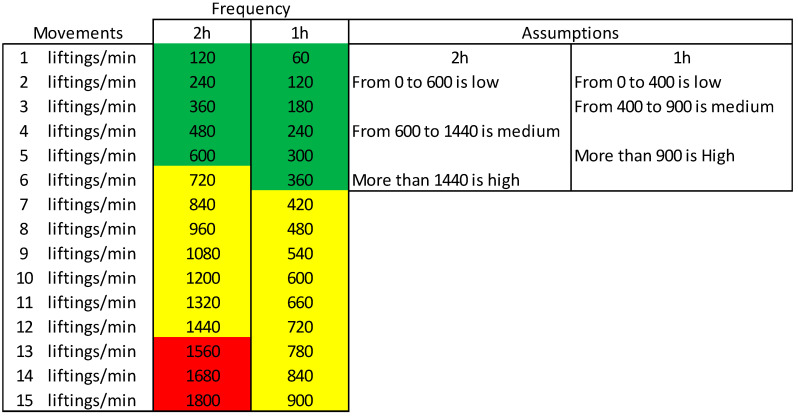
General assumptions for frequency for Table 3 and Table 7. The color green represents low risk, the color yellow represents medium risk and the color red represents high risk.

Assumptions for Table 6: as was mentioned in Section 2.3.2, Figure A1 from ISO 11228-1 standard and Table 1 from the NOM 036-1:2018 were combined to define the sets of parameters within the FzEA. During the fuzzification phase in Table 6, we more carefully divided the parameters; for example, both standards define its application over 3 kg, then we define low risk between 0 kg and 3 kg and low/medium risk in a range of 7 kg to 10 kg. The fuzzification was in the middle between 3 kg and 10 kg; the area between 7 kg and 10 kg is the critical zone for decisions; for medium risk, it should be located between 7 kg and 15 kg. Then, we chose to use a range of 8 kg to 13 kg; thus, the process of fuzzification started at 7 kg and finished at 13 kg. Finally, medium/high risk started at 13 kg, and from 15 kg to 25 kg or more, the risk was high (see Figure 5).

##### Lifting_Frequency

**Figure 6:**
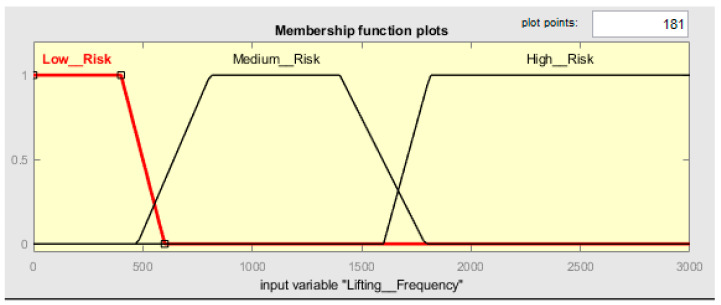
Membership function plots of Lifting_Frequency.

For the input variable called Lifting_Frequency, only 3 h of exposition time was considered, because over 3 h exceeds the frequency recommended when there is repetitiveness in tasks. The ideal parameters are between 5 kg and 10 kg with a frequency of 15 movements in 1 h. The membership of three trapezoidal fuzzy sets has been built considering the information presented in Table 3. The frequency below 400 movements has a membership set to one and, with all certainty, is a low risk (see Figure 6). To create a trapezoidal membership function, creating a decreasing ramp between the 400 movements to 600 movements is necessary to gradually smooth the degree of belonging in a range from 0 to 1, creating a transition from low risk to medium risk. The frequency above 1800 movements is certainly a high risk; it has a membership set to 1. To create a trapezoidal membership function, it is necessary to build an increasing ramp between 1600 movements and 1800 movements to gradually increase the degree of belonging in a range from 0 to 1. The frequency between 400 movements and 1600 movements should receive the same treatment, increasing the ramp between 400 movements and 800 movements and decreasing the ramp between 1400 movements and 1800 movements. The frequency between 400 movements and 600 movements and 1600 movements and 1800 movements represents the vagueness of the decision. Table 7 shows the risk ranges considered within the respective fuzzy sets.

General assumptions for frequency in Table 3 and Table 7 were made; The ISO 11228-1 recommends a maximal cumulate mass of 10,000 kg by shift and defines 15 lifting/min as maximum equivalent to 900 lifting in 1 h or 12 lifting/min equivalent to 1440 lifting in 2 h. Using Figure A1 in Appendix C, we define the risk for each lifting frequency. 

##### Work_Conditions

**Figure 7:**
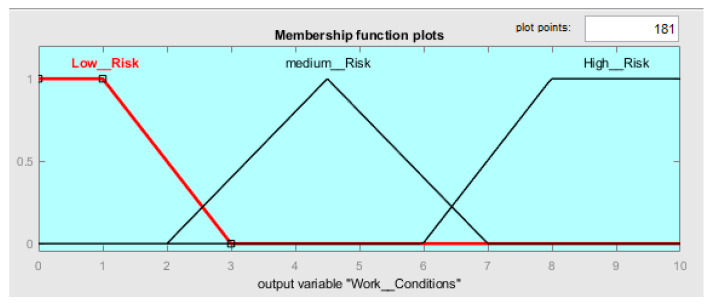
Membership function plots of Work_Conditions.

The output variable Work_Conditions was determined in a range of 0 to 10 points. The range from 0 to 3 represents fuzzy boundaries for low risk, the range from 2 to 7 represents fuzzy boundaries for medium risk, and the range from 6 to 10, represents fuzzy boundaries for high risk; see Figure 7. Table 8 shows the risk ranges considered with the respective fuzzy sets. A difference with input variables is that only two fuzzy sets are trapezoidal: the low and high risks. The medium-risk has a triangular shape.

The FzEA will determine the severity of the work-related health problem with a new category of results in a range of 0 to 10 points (see Table 8). Where a range from 0 to 3 represents a low risk of developing work-related illness over a long time, a range from 2 to 7 represents a risk of developing work-related illness in medium time; however, in this case, joint and muscular pain is occasionally present in most of the cases. Finally, the range from 6 to 10 represents a high risk of developing work-related illness in a short time; joint and muscular pain is present for long periods in most cases.

#### 2.5.2. Rules Definition

**Figure 8:**
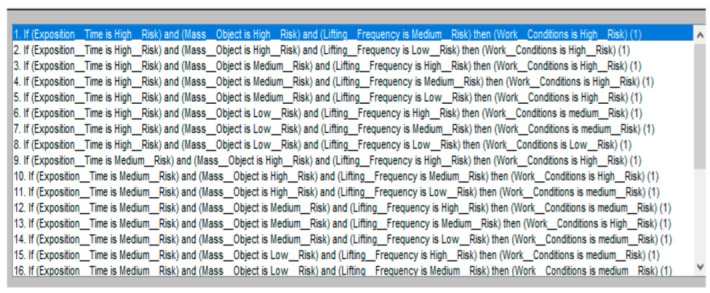
Fuzzy rules of decision.

The 27 rules of the decision were entered. They had the objective of simulating the opinion of experts or ergonomic analysts. An extract of the rules is depicted in Figure 8. 

#### 2.5.3. Defuzzification

Defuzzification is a process that combines the fuzzy set and the aggregation and produces an output in the form of a scalar number. Its value depends on the range of values assigned to the output variable, which represents the risk level assigned. The input variables have functions in a trapezoidal shape. The y-axis represents a probability range between 0 to 1. The x-axis represents the universe of discourse.

## 3. Results

**Figure 9:**
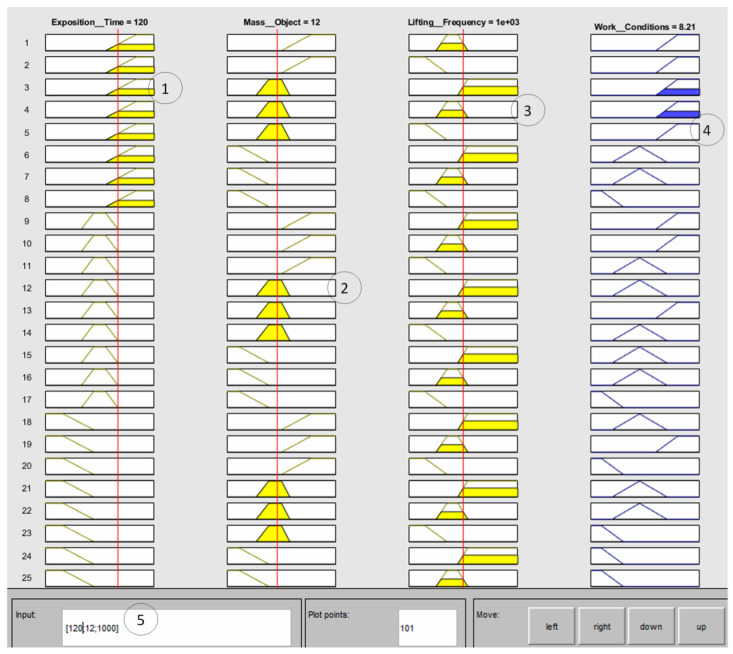
An example of the result showed in the Rule Viewer window. The highlighted rules are those applying to a specific work condition (1) High_Risk for Exposition_Time; (2) Medium_Risk for Mass_Object; (3) between Medium_Risk and High_Risk for Lifting_Frequency; (4) 8.21 for Work_Condition equivalent to High_Risk; (5) parameters entered 120 min, 12 kg, 1000 lifts.

Once the parameters and fuzzy sets were entered, the fuzzy inference for evaluating risk conditions FzEA (see Figure 9) was ready to be tested and validated. The aim of assessing manual material handling is to identify the non-ergonomic work conditions associated with an ergonomic level of risk (low, medium, or high). It is important to consider that the risk is always present. However, if it can be controlled and established as low risk, it is possible to minimize the impact on the workers’ health. As it was defined above, for this investigation, only three of them were considered. The elements selected as input variables were:

- The total time duration of the manual material handling in one shift, with 3 h maximal exposition time;
- The mass of the object to be manipulated, considered as maximal mass reference, which should never exceed 25 kg;
- The repetitiveness of the manual material handling task throughout the shift, considering that the maximal frequency of four lifts per min (1800 in 450 min of one shift) should never be exceeded.

The variables associated with the three fuzzy choices were:

- Low risk; does not generate work-related illness over a long period of time.
- Medium risk; generates work-related illness over a medium period of time.
- High risk; generates work-related illness over a short period of time.

The process of testing and validation were defined in two stages.

1. The testing stage comprised of feeding random data and verifying if the results obtained were according to the expected results.
2. The validation stage consisted of comparing the results from the fuzzy interface concerning results obtained from ergonomic assessments directly using the ISO 11228-1, referred to in the testing as “expected results”.

For the testing, 135 combinations of work conditions were evaluated through the 27 rules defined previously. The data were entered using the Ruler Viewer (see Figure 9). Some of the testing results are shown in Table 9, and all results are presented in Appendix A, which includes a combination of the three variables, the expected results (defined previously by the authors), and the Work_Conditions as numerical results of the fuzzy evaluation. Only results 13, 14, and 17 were different concerning expected results; however, this does not mean that the results are wrong. On the contrary, it more adequately locates the result of the combination of variables that are within decision limits. For example, in test 13, the exposition time of 119 min is closer to the high level than the medium level; therefore, the risk of 7.04 is on the border between medium and high-risk levels. In test 17, although the mass of the object is low (priority by standard), the exposition time and the frequency are medium risks. We can assume that the ergonomic work conditions were predicted with high precision. Consequently, FzEA can be used during a simple risk assessment when the evaluation depends mainly on analysts’ expertise and point of view, helping during the decision-making, as well as during detailed risk assessment, which includes a standardized method of evaluation.

## 4. Discussion

This work proposes a fuzzy logic ergonomic assessment (FzEA) as a decision support system (DSS) built in the MATLAB Fuzzy Logic Designer. The FzEA evaluates risk conditions present in tasks of manual material handling, which can lead to musculoskeletal disorders. The fuzzy interface is an interactive computer system, that helps specialists to solve differences in decision-making during ergonomic evaluations. The aim is to reduce the vagueness of the results using fuzzy logic applied in ergonomic decisions.

To define the fuzzy sets, three conditions of manual handling tasks were considered: the mass of the object to be manipulated; the duration of the task, defined as the time of exposition; and the lifting frequency. Even though the reference mass in the ISO 11228-1 includes different populations—from children to the elderly, the general domestic and general working population, and specialized workers—we could use all the range of reference mass included in it because we were limited by Table 1 of the Mexican standard NOM 036-1:2018, the content of which is quite different. So, the assumptions were oriented to comply with Mexican law, despite a combination of both standards being made within the FzEA. The standard ISO 11228-1 recommends that the limits of maximal mass reference and frequency must have the highest priority, and this priority was included in the fuzzy rules. However, it is important to consider that, despite a low-risk level being defined individually for each condition, the combination of different levels of risk could change the severity of the health impact. For example, in Table 10, results from FzEA show that despite 5 kg being considered low risk, its impact on the worker’s health could lead to a musculoskeletal disorder in a medium amount of time if other risk levels are present in the task.

These kinds of results are very valuable because the task evaluation in addition to the risk level includes the impact on the health, helping during the decision-making regarding whether or not to redesign a workstation. A case in which the three parameters are low is unlikely. However, if applicable, the task will be identified as safe. To manage all these conditions, a total of 27 inference rules have been developed, mathematically formulated to allow the conversion of the fuzzy system output into a single value, attributed to the work risk conditions. 

To verify the correct behavior of the system, results of 135 direct ergonomic assessments were considered. Then, some tests were carried out, considering parameters for different conditions of manual material-handling tasks. The comparison between the results from the direct assessments and the results from the FzEA differ in only three cases. Therefore, under the assumptions stated, the results are as expected. However, this first design phase of the FzEA was defined for 1 m of carrying distance, limiting the scope of the risk assessment; this important parameter is the base for determining the cumulated mass by shift. Consequently, a second design phase is required; this implies that Table 7 must be completed, considering the cumulated mass, and new assumptions about gender and age should be added. The project of the FzEA is the beginning of automation in decision-making during ergonomic interventions and lays the foundations for continually improving the interface, adding a greater number of fuzzy rules. This also includes other kinds of environmental risks. 

## 5. Conclusions

In this work, we propose a fuzzy logic ergonomic assessment (FzEA), as a decision support system (DSS) built in the MATLAB Fuzzy Logic Designer. Its objective is to evaluate tasks that include manual material handling and define the level of risk and the severity of the impact on the workers’ health. To manage all these conditions, a total of 27 inference rules were developed and mathematically formulated to allow the conversion of the fuzzy system output into a single value for work risk conditions. Accepting and adopting a method such as this would make it possible to unify the criteria when evaluating working conditions and help during the decision-making regarding whether or not to redesign a workstation, saving time and cost in each evaluation of risk. This first design phase of the FzEA was defined for 1 m of carrying distance, limiting the scope of the risk assessment; therefore, extending the number of linguistic variables would be an option to evaluate to analyze if it improves the resolution of the method. Making an App for mobile devices would allow this evaluation to be carried out everywhere.

## Figures and Tables

**Table 1:** Assignment of risk level for the time of exposition.

	Time of Exposition min
Low	0–80
Medium	60–120
High	100–180 or more

**Table 2:** Assignment of risk level for the mass of the object to be manipulated.

Risk Level	Mass of the Object kg
Low	0–10
Medium	7–15
High	13–25 or more

**Table 3:** Assignment of risk level for the frequency of manipulations.

Risk Level	Frequency of Carrying and Lifting Movements
Low	0–700
Medium	600–1100
High	900–1800 or more

**Table 4:** Fuzzy rules.

IF Time of Exposition	AND	IF Mass of the Object	AND	IF Frequency of Carrying and Lifting	THEN	The Risk Level of the Work Conditions
High		High		High		High risk
High		High		Medium		High risk
High		High		Low		High risk
High		Medium		High		High risk
High		Medium		Medium		High risk
High		Medium		Low		High risk
High		Low		High		High risk
High		Low		Medium		Medium risk
High		Low		Low		Low risk
Medium		High		High		High risk
Medium		High		Medium		High risk
Medium		High		Low		Medium risk
Medium		Medium		High		Medium risk
Medium		Medium		Medium		Medium risk
Medium		Medium		Low		Medium risk
Medium		Low		High		Medium risk
Medium		Low		Medium		Medium risk
Medium		Low		Low		Low risk
Low		High		High		Medium risk
Low		High		Medium		Medium risk
Low		High		Low		Low risk
Low		Medium		High		Medium risk
Low		Medium		Medium		Medium risk
Low		Medium		Low		Low risk
Low		Low		High		Low risk
Low		Low		Medium		Low risk
Low		Low		Low		Low risk

**Table 5:** Ranges of time of exposition and their fuzzy sets.

Variable	Fuzzy Set	Min
Exposition_Time	Low	0–40
	Low/Medium	60–80
	Medium	80–100
	Medium/High	100–120
	High	150 or more

**Table 6:** Ranges of the mass of the object and their fuzzy sets.

Variable	Fuzzy Set	kg
Mass_Object	Low	0–3
	Low/Medium	7–10
	Medium	8–13
	Medium/High	13–15
	High	20 or more

**Table 7:** Ranges of frequency of lifting and their fuzzy sets.

Variable	Fuzzy Set	Movements
Lifting_Frequency	Low	0–400
	Low/Medium	400–600
	Medium	600–1400
	Medium/High	1400–1800
	High	1800 or more

**Table 8:** Ranges of Work_Conditions and their fuzzy sets.

Variable	Fuzzy Set	Movements	Severity of the Risk
Work_Conditions	Low	0–1	No symptoms
	Low/Medium	1–3	Occasional pain in muscles and joints
	Medium	2–4.5	Frequent pain in muscles and joints
	Medium/High	4.5–7	The pain is present for long periods
	High	8 or more	

**Table 9:** Comparison of expected results concerning fuzzy interface results.

Test No.	Exposition_Time	Mass_Object	Lifting_Frequency	Expected Results	Work_Conditions
1	180	25	1800	HIGH	8.47
2	170	20	800	HIGH	8.21
3	121	17	400	HIGH	8.22
4	80	13	1000	MEDIUM	4.5
5	175	11	950	MEDIUM	8.36
6	165	8	625	MEDIUM	5.54
7	150	6	1700	MEDIUM	4.5
8	177	4	750	MEDIUM	4.5
9	110	2	500	LOW	1.24
10	115	23	1600	HIGH	8.17
11	90	21	700	HIGH	8.46
12	70	16	400	MEDIUM	3.77
13	119	9	1500	HIGH	7.04
14	95	12	850	MEDIUM	8.47
15	73	8	600	MEDIUM	5.3
16	65	7	1400	MEDIUM	3.17
17	80	5	650	LOW	4.50
18	115	3	300	LOW	1.33
19	80	22	1300	HIGH	8.47
20	75	19	900	HIGH	8.37
21	60	15	200	LOW	1.34
22	53	10	1200	MEDIUM	4.5
23	50	8	800	MEDIUM	3.34
24	45	14	200	LOW	1.24
25	35	1	1800	LOW	1.09
26	20	3	1000	LOW	1.22
27	15	5	500	LOW	1.24

**Table 10:** Severity of the risk for 5 kg mass for different exposition times and lifts.

Exposition Time	kg	Lifts	Risk Level	Severity of the Risk
34	5	150	Low	1.15
79	5	450	Low	1.5
88	5	2000	Medium	4.5
140	5	550	Medium	3.25
149	5	2000	Medium	4.5

## Data Availability

The dataset generated and analyzed during the present research is included in Appendix A.

## Abbreviations

GRE	General risk estimation.
DRE	Detailed risk estimation.
FL	Fuzzy logic.
ED	Ergonomic design.
EI	Ergonomic intervention.
FzEA	Fuzzy Ergonomic Assessment.
DDS	Decision support system.
FLD	Fuzzy logic designer.
FIS	Fuzzy interface system.

## Appendix A

**Table A1:** Results of comparison between direct assessment (Expected_results) and Fuzzy Ergonomic Assessment (Work_Conditions).

Test No.	Exposition_Time	Mass_Object	Lifting_Frequency	Expected Results	Work_Conditions
1	180	25	2700	HIGH	8.47
2	179	13	2500	HIGH	8.47
3	178	14	2300	HIGH	8.26
4	177	15	2100	HIGH	8.16
5	176	16	1900	HIGH	8.22
6	175	17	2160	HIGH	8.29
7	174	18	1900	HIGH	8.35
8	173	19	1750	HIGH	8.41
9	172	18	1140	HIGH	8.35
10	171	17	900	HIGH	8.29
11	170	16	535	HIGH	8.22
12	169	15	325	HIGH	8.16
13	168	14	497	HIGH	8.26
14	167	25	224	HIGH	8.47
15	166	14	67	HIGH	8.26
16	165	14	2400	HIGH	8.26
17	164	10	2100	HIGH	8.47
18	163	14	1800	HIGH	8.26
19	162	15	1600	HIGH	8.16
20	161	7	1300	MEDIUM	4.50
21	160	15	1518	HIGH	8.16
22	159	8	800	MEDIUM	6.35
23	158	14	2094	HIGH	8.26
24	157	13	950	HIGH	8.47
25	156	8	1647	MEDIUM	6.35
26	155	7	520	MEDIUM	2.48
27	154	15	895	HIGH	8.16
28	153	14	537	HIGH	8.17
29	152	14	685	HIGH	8.26
30	151	8	722	MEDIUM	6.35
31	150	2	2300	MEDIUM	4.50
32	149	5	2000	MEDIUM	4.50
33	148	1	1700	MEDIUM	4.50
34	147	4	1500	MEDIUM	4.50
35	146	3	1200	MEDIUM	4.50
36	145	2	1150	MEDIUM	4.50
37	144	7	1761	MEDIUM	4.50
38	143	7	1600	MEDIUM	4.50
39	142	6	800	MEDIUM	4.50
40	141	1	900	MEDIUM	4.50
41	140	5	550	LOW	3.25
42	139	4	400	LOW	1.13
43	138	2	385	LOW	1.14
44	137	3	220	LOW	1.14
45	136	7	300	LOW	1.27
46	119	25	2200	HIGH	8.20
47	118	13	1900	HIGH	7.26
48	117	14	1600	HIGH	8.16
49	116	15	1400	HIGH	8.16
50	115	16	1100	HIGH	8.17
51	114	17	1770	HIGH	8.17
52	113	18	1740	HIGH	8.19
53	112	19	1710	HIGH	8.21
54	111	18	1680	HIGH	8.20
55	110	17	1650	HIGH	8.19
56	109	16	575	MEDIUM	6.94
57	108	15	400	MEDIUM	5.68
58	107	14	555	MEDIUM	6.23
59	106	25	545	MEDIUM	5.94
60	105	14	535	MEDIUM	5.68
61	104	13	2400	MEDIUM	4.88
62	103	10	2100	MEDIUM	4.78
63	102	9	1800	MEDIUM	5.26
64	101	15	1500	HIGH	8.16
65	100	7	1200	MEDIUM	4.50
66	99	10	1620	MEDIUM	7.44
67	98	8	1590	MEDIUM	6.35
68	97	14	1560	MEDIUM	8.26
69	96	9	1530	MEDIUM	7.50
70	95	8	1500	MEDIUM	6.35
71	94	7	530	LOW	2.74
72	93	15	515	MEDIUM	5.45
73	92	14	500	MEDIUM	4.95
74	91	14	485	MEDIUM	4.69
75	90	8	470	MEDIUM	3.40
76	89	2	2300	MEDIUM	4.50
77	88	5	2000	MEDIUM	4.50
78	87	1	1700	MEDIUM	4.50
79	86	4	1400	MEDIUM	4.50
80	85	3	1100	MEDIUM	4.50
81	84	2	1470	MEDIUM	4.50
82	83	7	1600	MEDIUM	4.50
83	82	4	1500	MEDIUM	4.50
84	81	6	1470	MEDIUM	4.50
85	80	1	1440	MEDIUM	4.50
86	79	5	450	LOW	1.15
87	78	4	430	LOW	1.10
88	77	2	410	LOW	1.10
89	76	3	390	LOW	1.12
90	75	8	370	LOW	3.40
91	74	25	2700	HIGH	7.64
92	73	13	2500	MEDIUM	4.50
93	72	14	2300	MEDIUM	5.26
94	71	15	2100	HIGH	6.57
95	70	16	1900	HIGH	6.87
96	69	17	1390	HIGH	8.24
97	68	18	1340	HIGH	8.21
98	67	19	1290	HIGH	8.19
99	66	18	1240	HIGH	8.17
100	65	17	1190	HIGH	8.17
101	64	16	350	LOW	2.96
102	63	15	330	LOW	2.85
103	62	14	310	LOW	2.34
104	61	25	290	LOW	1.89
105	60	14	270	LOW	1.28
106	59	14	2600	MEDIUM	4.50
107	58	10	2400	MEDIUM	4.50
108	57	14	2200	MEDIUM	4.50
109	56	15	2000	MEDIUM	4.50
110	55	9	2300	MEDIUM	4.00
111	54	15	1140	HIGH	8.16
112	53	8	1090	MEDIUM	3.40
113	52	14	1040	MEDIUM	5.26
114	51	13	990	MEDIUM	4.50
115	50	8	940	MEDIUM	3.40
116	49	7	250	LOW	1.27
117	48	15	230	LOW	1.34
118	47	14	210	LOW	1.24
119	46	14	190	LOW	1.24
120	45	8	170	LOW	1.31
121	44	2	2700	LOW	1.15
122	43	5	2500	LOW	1.15
123	42	1	2300	LOW	1.14
124	41	4	2100	LOW	1.13
125	40	3	1900	LOW	1.12
126	39	2	890	LOW	1.11
127	38	7	840	LOW	1.27
128	37	7	790	LOW	1.27
129	36	6	740	LOW	1.21
130	35	1	690	LOW	1.18
131	34	5	150	LOW	1.15
132	33	4	130	LOW	1.10
133	32	2	110	LOW	1.07
134	31	3	90	LOW	1.06
135	30	8	70	LOW	1.31

## Appendix B

**Table A2:** Reproduction of Table 1 maximal mass reference that can lift or down a worker, sorted by age and gender contained in the Mexican Standard NOM 036-1:2018 [6].

Mass Reference kg	Gender	Age (in Years)
7	Female	Under 18
	Male	
15	Female	Over 45
20	Female	Between 18 and 45
	Male	Over 45
25	Male	Between 18 and 45
